# Chronic Pectoralis Major Rupture Reconstruction With Interpositional Acellular Dermal Allograft

**DOI:** 10.31486/toj.20.0003

**Published:** 2021

**Authors:** Conor J. C. Gouk, Ryan M. Shulman, Christine Lowe, Craig Buchan, Michael J. E. Thomas, Fraser J. Taylor

**Affiliations:** ^1^ Department of Trauma and Orthopaedics, Gold Coast University Hospital, Southport, Queensland, Australia; ^2^ Griffith University, Southport Gold Coast, Queensland, Australia; ^3^ Department of Radiology, Gold Coast University Hospital, Southport, Queensland, Australia; ^4^ Department of Anaesthetics, Gold Coast University Hospital, Southport, Queensland, Australia

**Keywords:** *Allografts*, *pectoralis muscles*, *rupture*, *tendon injuries*, *tendons*

## Abstract

**Background:** Pectoralis major tendon (PMT) rupture commonly occurs in males 20 to 39 years of age. PMT rupture is most often associated with gym-based exercise, with attempted bench press being the most common causative event, but it is also associated with contact or impact sports. Delayed presentation, misdiagnoses, and chronic PMT rupture can result in a therapeutic dilemma.

**Case Series:** We present 2 cases of chronic PMT rupture that were operatively managed using acellular dermal allograft as an interposition graft. Patients’ final follow-ups were at 20 and 30 months, respectively. Strength in their pectoralis major muscle was well preserved on the contralateral side: 88% for patient 1 and 110% for patient 2.

**Conclusion:** Our reported technique using an interpositional acellular dermal allograft is a good option to treat chronic PMT rupture.

## INTRODUCTION

Pectoralis major tendon (PMT) rupture commonly occurs in males 20 to 39 years of age.^1^ PMT rupture is most often associated with gym-based exercise, with attempted bench press being the most common causative event, but it is also associated with contact or impact sports.^2^ Rupture is often associated with anabolic steroid use, which has a significant dysplastic effect on the collagen fibrils, thereby creating poor tendinous substance.^3^ The most common site of rupture is at the tendinous insertion^4^; however, cases of rupture have been reported along the whole tendon substance. The degree of injury and location of injury form the basis for the Tietjen classification: (1) contusion/sprain, (2) partial tear, (3) complete tear, (3A) muscle origin, (3B) muscle belly, (3C) musculotendinous junction, or (3D) tendon near insertion.^5^ Hanna et al have shown that injuries at the musculotendinous junction (3C per the Tietjen classification) are the most difficult to address and have the poorest outcomes.^6^ Delayed presentation, misdiagnoses, and chronic PMT rupture can result in a therapeutic dilemma.

Patients with acute PMT rupture experience pain, tenderness, dysfunction, ecchymosis, and bunching of the pectoralis major muscle; weakness and asymmetry/cosmetic disturbance of the chest wall are described in unrecognized chronic rupture. The most specific sign of PMT rupture is thinning of the anterior axillary fold.^1^ Ultrasound can be used when PMT rupture is suspected, but magnetic resonance imaging (MRI) appears to be the gold standard for imaging.^7^

Acute PMT rupture has previously been defined as occurring within a time period of less than 6 weeks, with chronic rupture defined as being more than 6 weeks old.^8^ Butt et al demonstrated in their review article that acute ruptures have many available techniques for primary repair, such as direct repair, transosseous suture fixation with or without bone trough, anchor fixation, and direct repair to clavipectoral fascia.^9^ However, if patients present late, retraction, poor tendinous substance, and the location of the tear can make repair or reconstruction of a chronic PMT rupture challenging. Direct repair techniques are often not possible. Techniques described for chronic PMT ruptures include primary repair using a variety of screws and washers, cortical buttons, and end-to-end suture with or without hamstring autograft augmentation, hamstring allograft, bone patella autograft, fascia lata allograft, and tendoachilles allograft.^1,3,10-14^

In the case of chronic/retracted PMT rupture and in tendon ruptures at other sites, acellular dermal allografts (ADAs) have been used successfully as an augmentation and as an interposition graft.^15-17^ The intended purpose of the ADA is for the graft to act as a scaffold for normal cellular integration. Using an ADA in an interpositional manner decreases the tension on the repair. High tension has been shown to be detrimental to successful tendon repair.^18^

We present 2 cases of chronic PMT rupture that were operatively managed using the GRAFTJACKET ADA (Wright Medical) as an interposition graft.

## CASE SERIES

### Patient 1 Presentation

A right-hand-dominant, 34-year-old male employed as an upholsterer presented to our orthopedic outpatient clinic via his general practitioner complaining of weakness and pain to his left shoulder 1 year after an injury that occurred while he was unloading a go-kart from the back of a truck. At the time of injury, his arm was forced into the classic position of injury: abduction and external rotation. On examination, the patient had obvious bunching of the pectoral muscle and thinning of the anterior axillary fold, and he was weak with shoulder adduction. Because of his weakness and the effect the injury had on his work and go-kart hobby, the patient was offered operative intervention.

### Patient 2 Presentation

**Figure 1.**
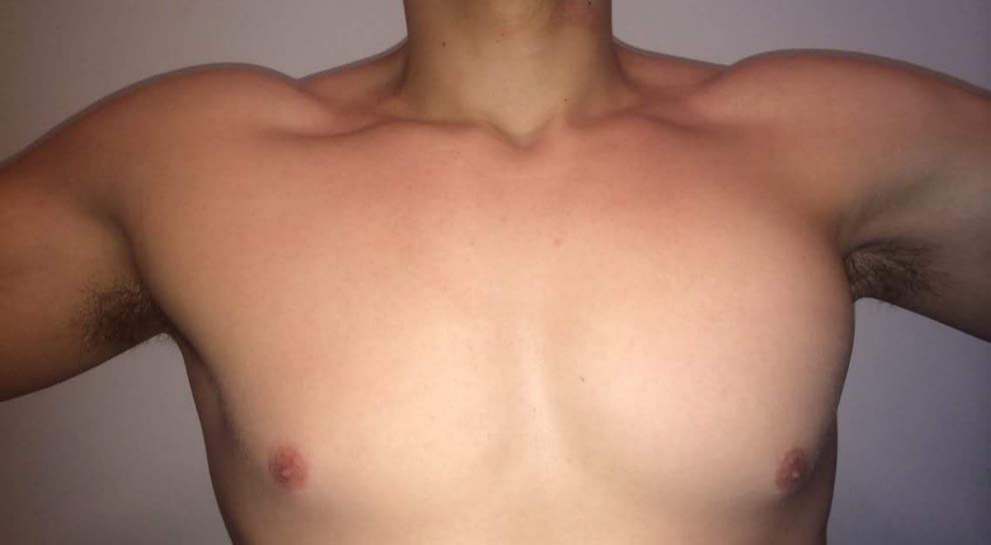
Preoperative photo of patient 2 shows asymmetry of the axillary folds; folds are thinned on the left with apparent bunching at the superolateral aspect of the pectoral region.

A right-hand-dominant, 21-year-old male employed as a fitter and turner presented to our orthopedic outpatient clinic via his general practitioner. He complained of some weakness but primarily of chest wall asymmetry and poor cosmesis to such a degree that he had sought the help of a psychologist 9 months after an injury to his left shoulder sustained while bench pressing. Examination demonstrated bunching of the pectoral muscle, thinning of the anterior axillary fold (Figure 1), and weak shoulder adduction. Because of the detrimental effect the injury was having on the patient's mental health and day-to-day activities, he was offered operative intervention.

### Assessment and Planning

**Figure 2.**
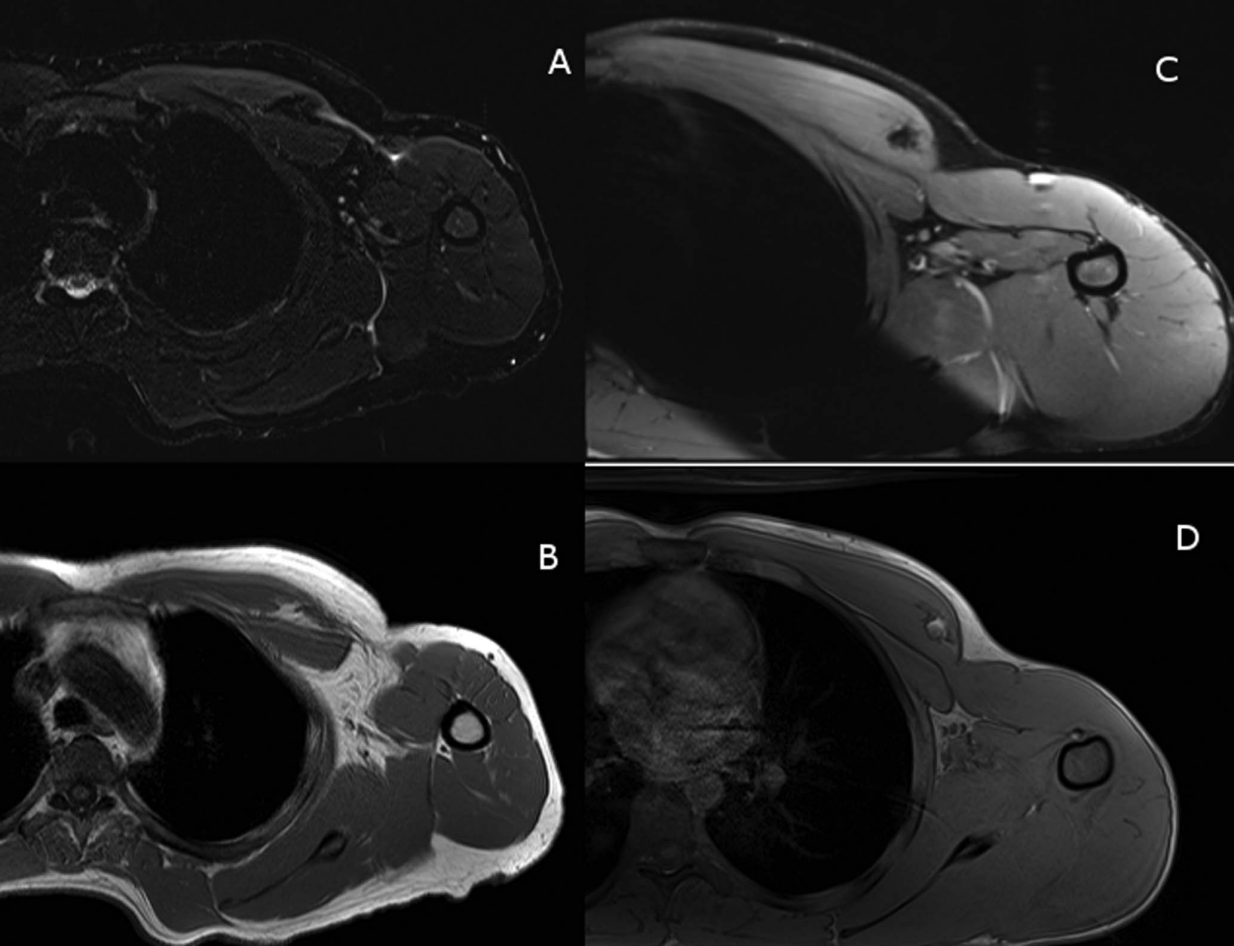
Preoperative magnetic resonance images. (A) Patient 1 axial proton-density-fat-saturation (PD-FS) image demonstrates pectoralis major tendon (PMT) rupture with retraction and a large fluid signal defect. (B) Patient 1 axial T1 image demonstrates tendon retraction and fat-filled defect in the deltopectoral groove. (C) Patient 2 axial PD-FS image demonstrates PMT rupture with marked retraction and bunching. (D) Patient 2 axial T1 image also demonstrates the rupture and confirms a small amount of peritendinous fat retracted with the tendon into the medially displaced myotendinous junction.

Both patients were nonsmokers, neither had any medical comorbidities, and both denied any form of steroid use. MRI scans were obtained for preoperative planning, and axial images are shown in Figure 2. For both patients, the location of the rupture was musculotendinous (3C per the Tietjen classification).^5^

For each patient, we secured authorization to use the GRAFTJACKET ADA (Wright Medical) product from the Australian Therapeutic Goods Administration through Special Access Scheme (SAS) Category B. The SAS allows health practitioners to access therapeutic goods that are not included in the Australian Register of Therapeutic Goods on a case-by-case basis**.**

### Surgical Technique

**Figure 3.**
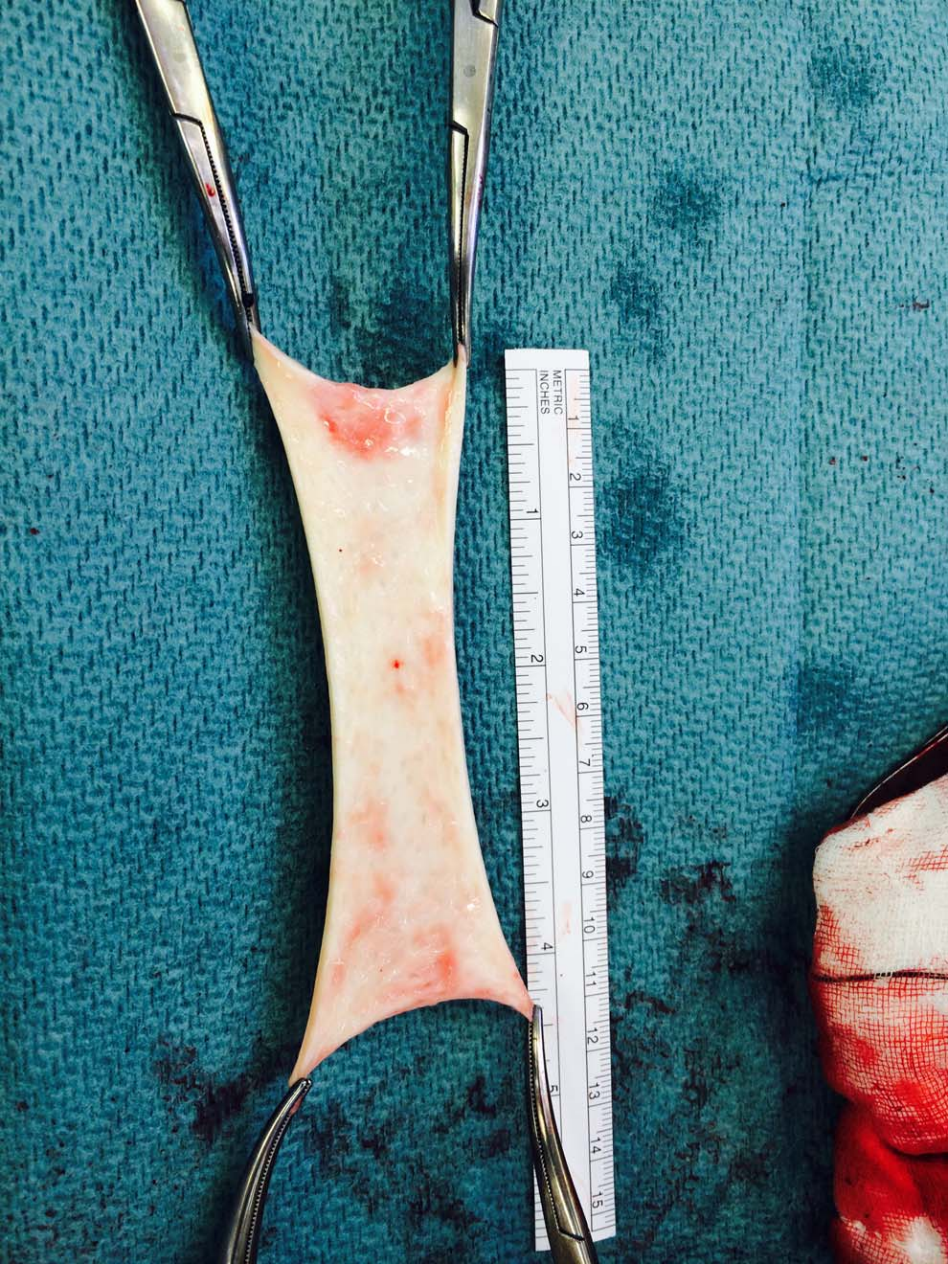
Technique for pretensioning the dermal allograft using 4 artery clips.

**Figure 4.**
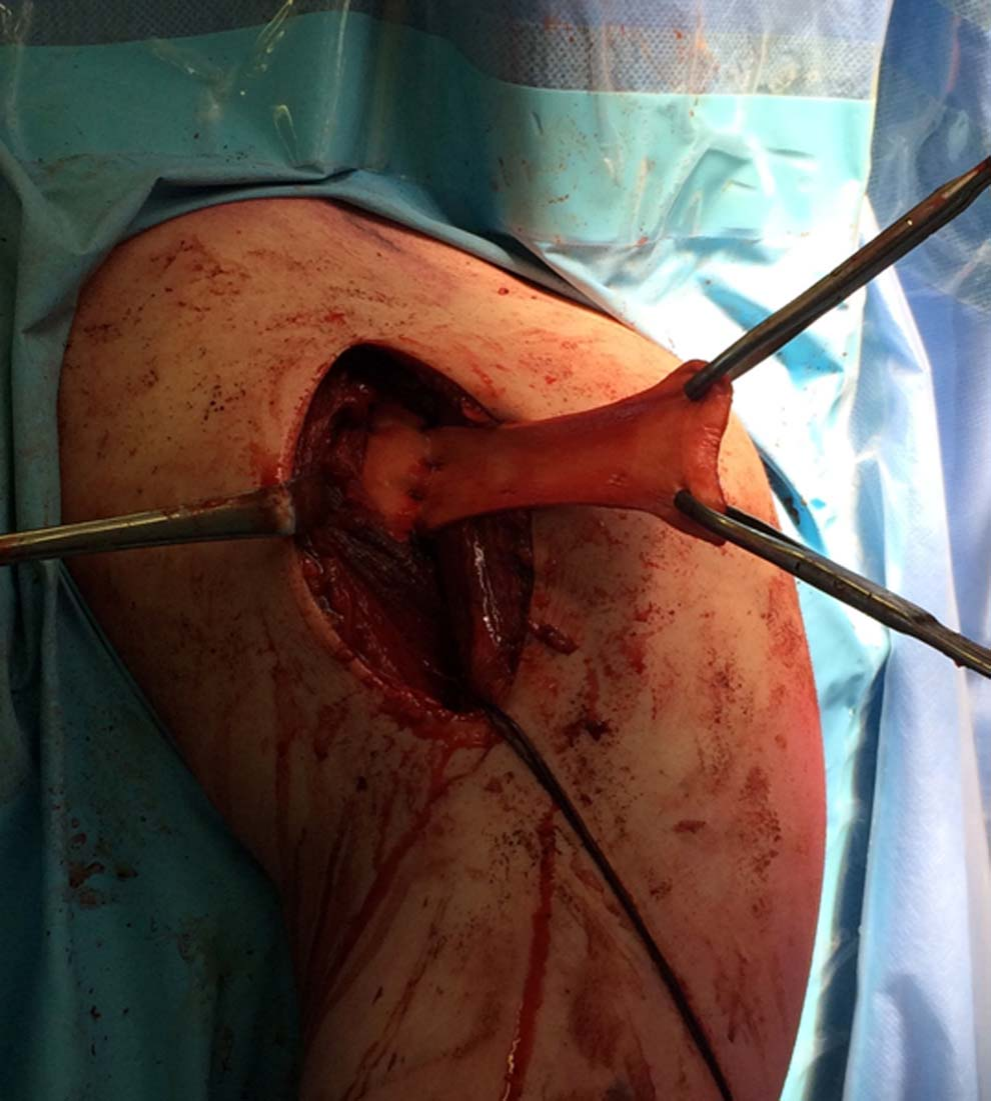
Acellular dermal allograft sutured medially to the ruptured pectoral musculotendinous junction.

**Figure 5.**
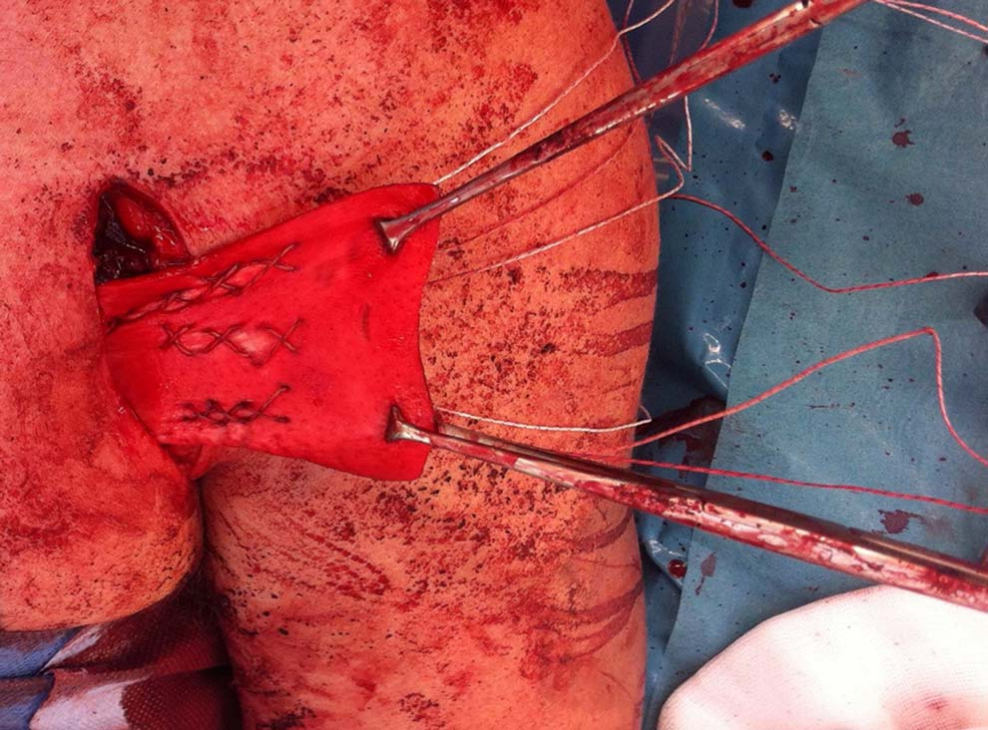
Anchors sutured to the acellular dermal allograft in a running cruciate/modified Becker fashion.

Under general anesthesia, the patients were placed in the beach chair position using the T-MAX Shoulder Positioner with pneumatic arm holder (Smith and Nephew). A 6-cm modified deltopectoral approach was used, advancing medially to identify the ruptured and retracted PMT. Care was taken to preserve the cephalic vein by retracting it laterally. In both patients, the clavicular portion of the pectoralis major was intact. Even at such chronic stages, evident in both patients, inflammatory exudate still surrounded the ruptured tendons, indicating their positions. The residual tendinous substances, medially and laterally, were dissected and mobilized as able to ensure that no tethering limited the tendinous stump mobility. To ensure safe dissection of the pectoralis major muscle and to reduce the risk of denervation, the surgeon approached the tendon from the inferior and lateral aspects with blunt dissection. Care was taken to develop a plane between the pectoralis major and minor muscles, reducing the risk to the lateral pectoral nerve and its branches. The defect was then fully assessed, and the decision was made to proceed with interposition grafting. The ADA was rehydrated and prepared per the manufacturer's instructions. The ADA was pretensioned using 2 pairs of artery clips (Figure 3). The graft was overlapped with the tendinous stump by 2 cm, dermal side facing away from the tendinous surface, and the medial free edge was sewn to the body of the ADA with multiple large, braided, nonabsorbable, interrupted sutures (#2 Force Fiber, Wright Medical). As shown in Figure 4, the ADA was superiorly sutured to the intact clavicular head tendon in a running fashion, using the same large, braided, nonabsorbable suture material. The graft was secured laterally to the humeral footprint of the PMT at the lateral aspect of the bicipital groove, using 3 PITON anchors (Tornier, Inc). The anchors were sutured to the ADA centrally and 0.5 cm from the lateral borders, using a running cruciate/modified Becker style^19^ (Figure 5). The intact clavicular head was a helpful reference to determine length and tension of the repair. The bicep tendon was protected throughout. A layered closure was performed, the wound was dressed, and a shoulder immobilizer was fitted.

### Rehabilitation and Follow-Up

Postoperatively, both patients had regular follow-up and carefully regulated rehabilitation. For the first 3 weeks postoperatively, patients were allowed to do pendular and passive shoulder range of motion (ROM) but were instructed to keep their arm in the sling between exercises. From 3 weeks, patients were to do isometric exercises and active-assisted ROM. From 6 weeks, patients progressed to rotator cuff strengthening supervised by a physiotherapist.

**Figure 6.**
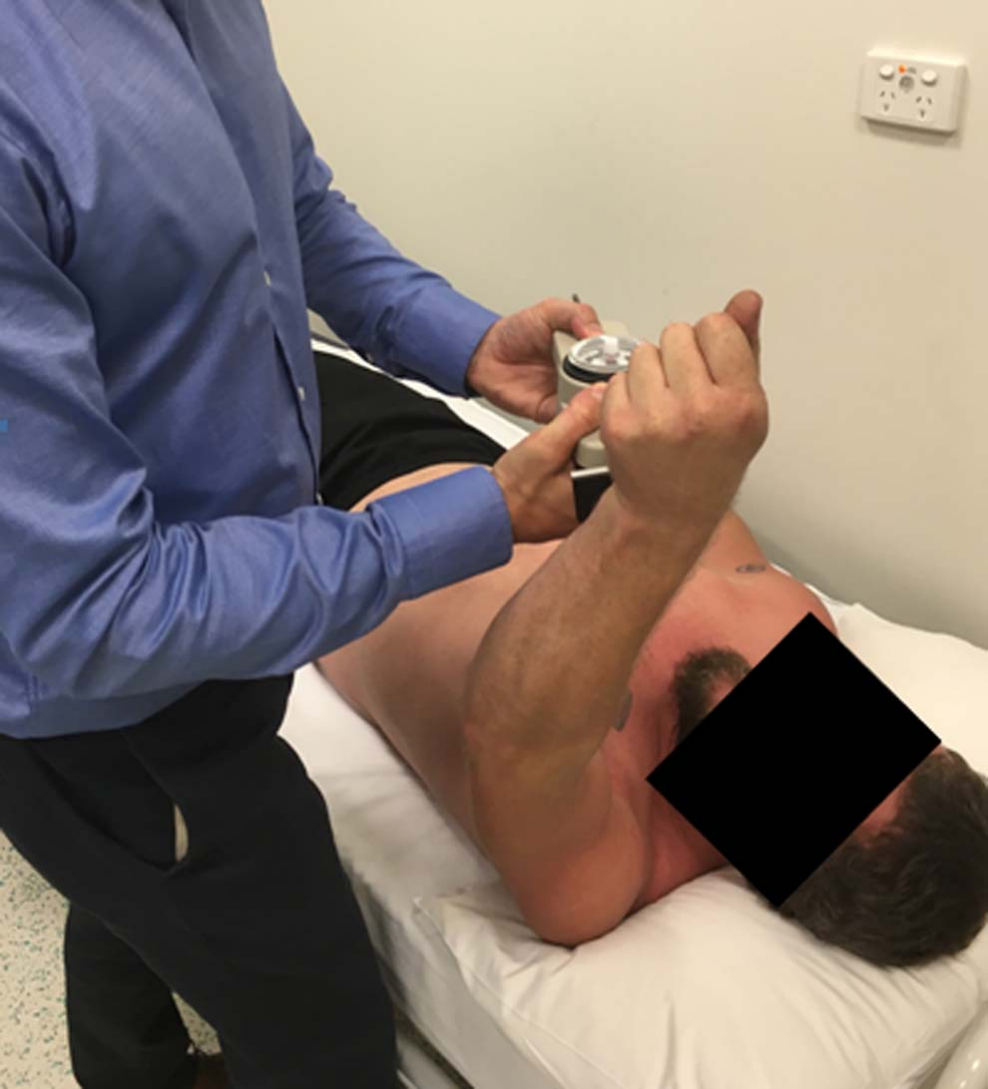
Method of assessment of pectoral strength using an analog hydraulic push-pull dynamometer.

At time of final follow-up, the patients completed the Disabilities of the Arm, Shoulder and Hand (DASH); Constant-Murley Shoulder; and Oxford Shoulder Score questionnaires and underwent a strength assessment of their pectoralis major muscle and rotator cuff using an analog hydraulic push-pull dynamometer (Baseline Evaluation Instruments). A mean of 3 attempts was recorded in pounds and expressed as a percentage of the contralateral side. Strength assessment of the pectoralis major muscle was carried out by positioning the subject supine with the shoulder forwardly flexed to 90 degrees and the elbow fully extended; the dynamometer was placed on the volar wrist crease, and adduction strength was measured as illustrated in Figure 6.

Both patients also had postoperative MRIs at time of final follow-up that were evaluated by 2 musculoskeletal fellowship–trained radiologists to assess graft integrity and muscle growth. Muscle growth was established by measuring muscle belly circumference at 3 predetermined sites preoperatively and postoperatively. These measurements were compared at each site and expressed as a percentage of growth.

## Results

Final follow-up was at 20 months for patient 1 and 30 months for patient 2. Strength in the pectoralis major muscle of the contralateral side was well preserved: 88% for patient 1 and 110% for patient 2. Rotator cuff strength was comparable to the contralateral side. Both patients were happy they had undergone surgery. Outcome scores are shown in Table 1.

**Table 1.** Functional Scores at Final Follow-Up

By Case and Overall	Constant-Murley Shoulder Score (0-100)	Oxford Shoulder Score (0-60)	DASH Score (0-100)
Patient 1	90	48.0	0.000
Patient 2	90	47.0	1.724
Mean	90	47.5	0.862

Notes: For the Constant-Murley Shoulder Score, the higher the score, the better the functionality. For the Oxford Shoulder Score, the lower the score, the better the functionality. For the DASH Score, the lower the score, the lower the disability.

DASH, Disabilities of the Arm, Shoulder, and Hand.

**Figure 7.**
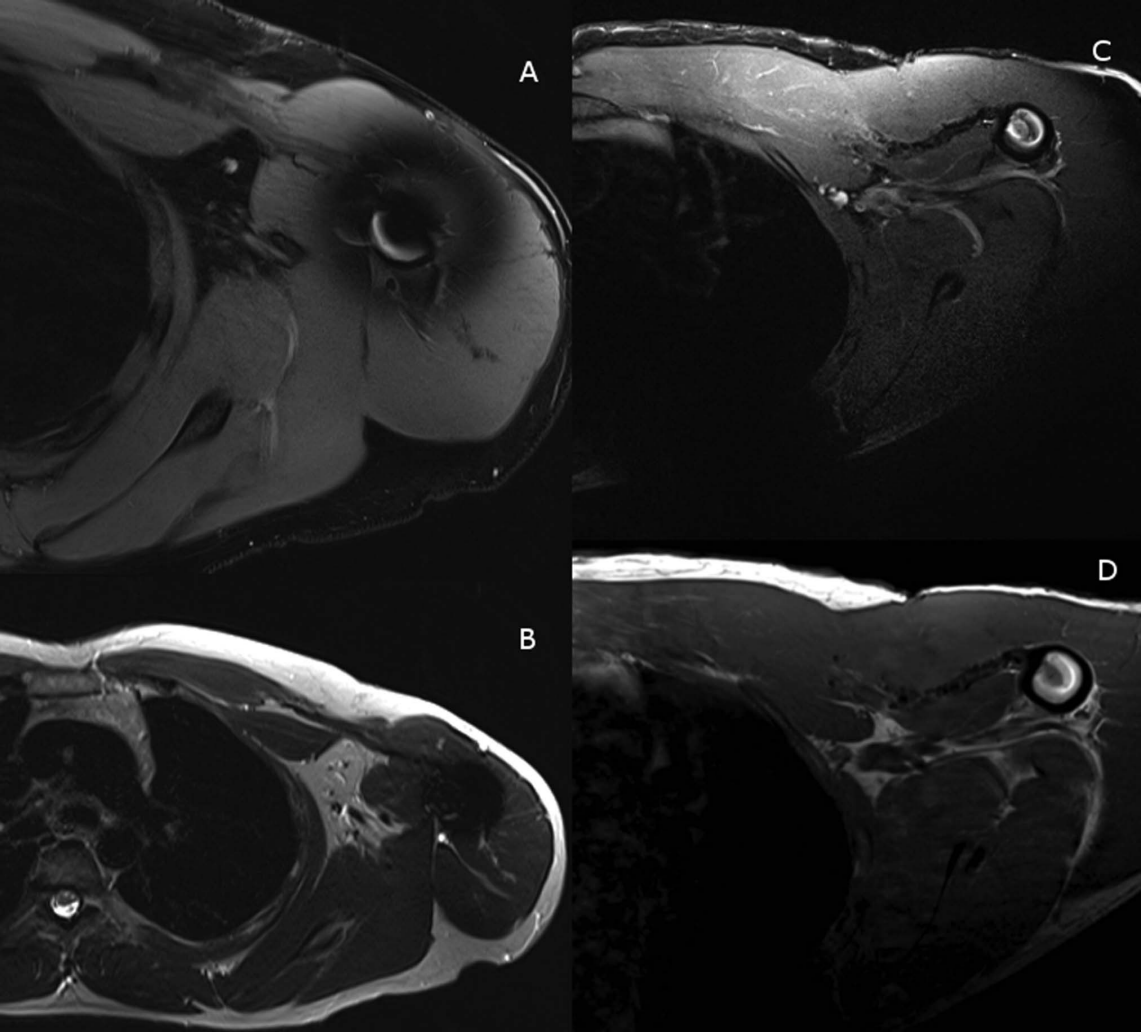
Postoperative magnetic resonance images. (A and B) Axial images for patient 1 demonstrate dermal allograft interpositional repair intact and in continuity. (C and D) Axial images for patient 2 demonstrate dermal allograft interpositional repair intact and in continuity.

**Figure 8.**
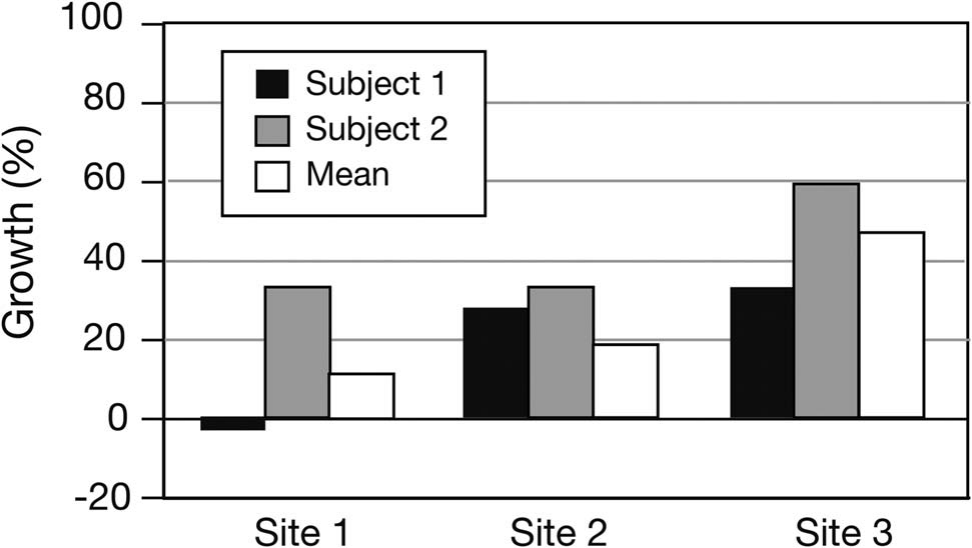
Percentage growth of pectoralis major muscle at 3 predetermined sites using preoperative and postoperative magnetic resonance imagining assessment and mean values.

MRI evaluation showed that the grafts were intact (Figure 7). Muscle growth, determined by measuring preoperative and postoperative muscle volume at the 3 predetermined sites, predominantly showed significant recovery (Table 2 and Figure 8). Site 1 was the lateral site, and patient 1 had a degree of atrophy at this site, likely related to its proximity to the surgical site.

**Table 2.** Percentage Postoperative Change of Cross-Sectional Volume of Pectoralis Major Muscle at Three Predetermined Sites Using Preoperative and Postoperative Magnetic Resonance Imaging Assessment and Mean Values

By Case and Overall	Site 1 (Lateral), %	Site 2 (Middle), %	Site 3 (Medial), %
Patient 1	–2	28.0	33.0
Patient 2	24	9.0	60.0
Mean	11	18.5	46.5

**Figure 9.**
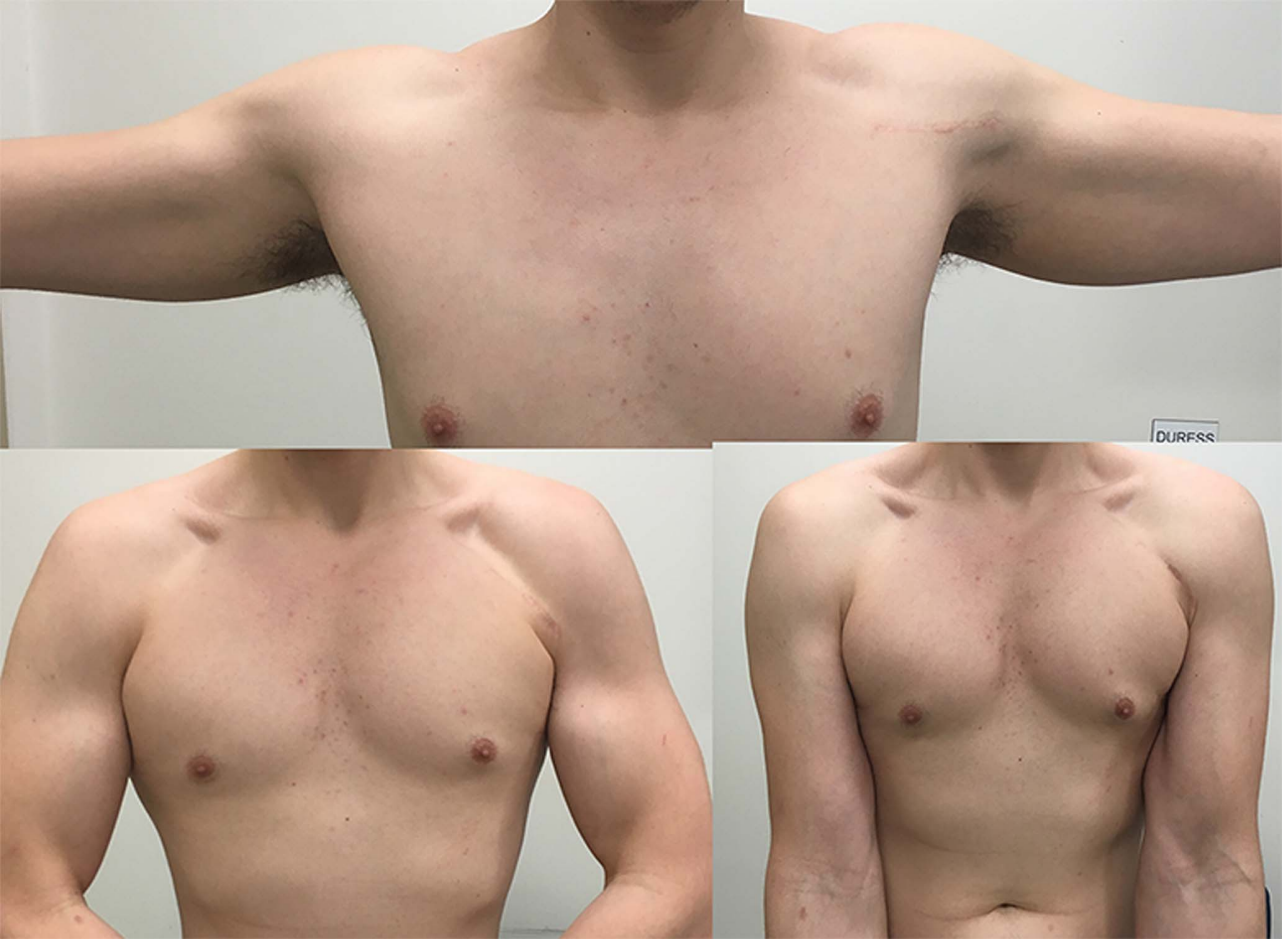
Postoperative photos of patient 2 show restoration of the symmetry of his anterior axillary fold and superolateral pectoral border and his surgical scar.

Twelve months postoperatively, patient 1 reported that he was back to work and go-karting without problem. Patient 2 reported that he was very happy with the cosmetic outcome (Figure 9), was back to work without problem, and could repetitively bench press a 45-kg barbell.

## DISCUSSION

The incidence of PMT rupture is increasing, and delayed diagnosis is common.^13,15^ Conservative treatment may be appropriate in the elderly population; however, younger patients who are in the peak incidence range do not tolerate the limitations posed by PMT rupture.^9,20-23^ Chronic ruptures are surgically problematic for many reasons and have been shown to have poorer outcomes than acute repairs.^24^

Because of the chronicity of the PMT ruptures surgically managed and reported in these 2 cases, the ADA was used in an interpositional fashion. In the experience of the senior author (F.J.T.), the described technique is preferred to a sleeve technique. The ADA technique has many benefits, including avoidance of donor site morbidity compared to autograft; avoidance of rejection; and reduced costs, obtainment and storage issues, and disease transmission compared to allograft.^25-28^ Disease transmission has not been reported, and the risk is described as theoretical.^28,29^

Neumann et al demonstrated that ADAs can be successfully used for the repair of the PMT as an augment.^15,20^ Dehler et al used the ADA as an interposition graft in a similar manner to our technique, with improvement in QuickDASH score, cosmesis, and strength.^15^

## CONCLUSION

Because of the results of this case study and the benefits of the ADA, we feel that using an interpositional ADA in our reported technique is a good option to treat chronic PMT rupture.

## Notes

*This article meets the Accreditation Council for Graduate Medical Education and the American Board of Medical Specialties Maintenance of Certification competencies for Patient Care and Medical Knowledge.*
